# Altered Gut Microbiota in H1-Antihistamine-Resistant Chronic Spontaneous Urticaria Associates With Systemic Inflammation

**DOI:** 10.3389/fcimb.2022.831489

**Published:** 2022-03-16

**Authors:** Yao Song, Kena Dan, Zhengqiu Yao, Xi Yang, Bangtao Chen, Fei Hao

**Affiliations:** ^1^Department of Pediatrics, The Third Affiliated Hospital of Chongqing Medical University, Chongqing, China; ^2^Department of Dermatology, The Third Affiliated Hospital of Chongqing Medical University, Chongqing, China; ^3^Department of Dermatology, Chongqing University Three Gorges Hospital, School of Medicine, Chong University, Chongqing, China

**Keywords:** gut microbiota, chronic spontaneous urticaria (CSU), antihistamine resistance, inflammation, 16S rRNA sequencing

## Abstract

**Background and Objective:**

Chronic spontaneous urticaria (CSU) is a histamine-mediated inflammatory skin disease, and second-generation non-sedating H1-antihistamines (nsAH) at licensed doses have long been the first-line therapy in CSU. However, about 50% of patients are resistant to nsAH, and the precise pathogenesis remains largely unknown but seems to be associated with low-level systemic or intestinal inflammation. We aim to determine the fecal microbial composition and clarify its correlation with the clinical profiles og CSU with nsAH resistance.

**Methods:**

A total of 25 CSU patients with or 19 CSU patients without nsAH resistance and 19 healthy controls (HC) were enrolled in this study. The intestinal microbiome was detected by 16S rRNA sequencing. The data were analyzed using *R* language software.

**Results:**

Significantly higher urticarial activity score for 7 days, stool calprotectin, erythrocyte sedimentation rate, serum C-reactive protein, and interleukin-6, but much lower alpha-diversity and evenness of fecal bacterial community were observed in CSU patients with nsAH resistance than in those without (*P* <0.05 for all variables). Compared to patients with nsAH-responsiveness, the abundance of fecal genera *Prevotella*, *Megamonas*, and *Escherichia* were significantly increased, while that of *Blautia*, *Alistipes*, *Anaerostipes*, and *Lachnospira* were remarkably reduced in nsAH-resistant patients (uncorrected *P* <0.05 for all variables). Finally, systemic not intestinal inflammation degree was positively correlated with genera *Escherichia*, while negatively with genera *Blautia*, *Dorea*, *Lactobacillus*, *Eubacterium_hallii_group*, and *Roseburia*. CSU without nsAH resistance and HC individuals showed almost unchanged genera bacterium.

**Conclusions:**

Among CSU patients, pro-inflammation phenotype relating to enteric dysbacteriosis features nsAH resistance in CSU patients. The results provide clues for future microbial-based or anti-inflammatory therapies on nsAH resistant CSU.

## Introduction

Whether a spontaneous or inducible subtype, chronic urticaria is characterized by recurrent episodes of pruritic wheals with or without associated angioedema persisting longer than 6 weeks (Zuberbier et al., 2018). Globally, up to 1% of the general population suffers from chronic urticaria at some point in life, and a trend of increasing prevalence has been uncovered over recent years (Gonçalo et al., 2021). In dermatologic practice, the disease prevails in women aged 30 to 50 years, and chronic spontaneous urticaria (CSU) accounts for about two-thirds of all chronic urticaria cases (Antia et al., 2018). CSU, especially related angioedema, causes remarkably negative impacts on work productivity and daily activities due to the co-existence of anxiety and psychological distress and frequent medical needs (Gonçalo et al., 2021).

CSU is generally believed to be a histamine-mediated allergic reaction, and second-generation non-sedating H1-antihistamines (nsAH) at licensed doses have long been the first-line therapy in CSU. Clinically, resistant CSU (RCSU) is defined by the patients who are unresponsive to any nsAH at approved dosage after 2 to 4 weeks of treatment, and RCSU accounts for 40 to 60% of patients receiving the standard treatment (Maurer et al., 2017). The mechanism of nsAH resistance remains largely unknown, and thus the issue is currently an important and troublesome matter in the clinical management of CSU. With the property of antagonizing proinflammatory reaction, the modalities, namely, increasing doses (up to 4-fold of the approved dose) of nsAH, glucocorticoids, or cyclosporine are alternative therapeutics for RCSU (Min and Saini, 2019). Therefore, inflammation is largely involved in the occurrence of nsAH resistance in CSU (Bansal and Bansal, 2019; Min and Saini, 2019).

Together with other colleagues, we previously proved that inflammation abnormalities mediated by the imbalance of Th1/Th2/Th17 cytokines might contribute to CSU pathogenesis, and thus, understanding of the possible sources of inflammation in real-world settings is critical in enabling measures to improve symptom control and minimize disease burden among CSU patients (Chen et al., 2018; Bansal and Bansal, 2019). Inflammation abnormalities could be caused by alterations in the gut microbiome (Al Bander et al., 2020), and recent evidence from human metabolomics suggested enteric dysbacteriosis in CSU cases compared to the healthy cases (Lu et al., 2019; Wang et al., 2020; Liu et al., 2021: Wang et al., 2021; Zhang et al., 2021). The assumed importance of gut microbiota in the pathogenesis of CSU also can be evidenced by the finding that it was involved in differentiation and maturation of T lymphocytes, inflammasome activation, and emotional regulation (Gensollen et al., 2016; Yao et al., 2017; Chen, 2017; Man, 2018; Zhou et al., 2019). However, the gut microbiota profile in RCSU patients and its link with systemic or intestinal inflammation states failed to be clarified in previous works. The unresolved issues should deserve more attention as RCSU patients suffer from more serious social and psychological burdens. Herein, we hypothesize that pro-inflammation phenotype features nsAH resistance in CSU patients which may associate with enteric dysbacteriosis. The current study aims to determine the fecal microbial composition by 16S rRNA sequencing and clarify its correlation with the clinical profiles of CSU with nsAH resistance.

## Materials and Methods

### Study Population

This investigation was conducted in the dermatology clinic and physical examination department of a comprehensive tertiary hospital in Chongqing, China, from May to December 2020. Voluntarily, patients who had not taken antihistamines for at least 1 month before enrollment were administered levocetirizine (5 mg per day). By 2 weeks of follow-up, a total of 25 RCSU patients aged ≥18 years (RCSU group) and 19 age- and sex-matched CSU patients with an adequate response to levocetirizine (CSU group) were enrolled, and meanwhile, 19 age- and sex-matched healthy controls (HC group) were recruited. The diagnosis of CSU was made by board-certified dermatologists following the criteria proposed by the new EAACI/GA^2^LEN/WAO/EDF guidelines (Zuberbier et al., 2018). A CSU patient resistant to treatment with levocetirizine (5 mg per day, 2 weeks) was classified into the RCSU group. All participants have similar usual living and eating habits.

Exclusion criteria included: (a) pregnant or lactating women, (b) patients with previous or current allergic and autoimmune diseases, systemic diseases (such as inflammatory bowel diseases and metabolic diseases), or skin diseases other than urticaria (such as acne, atopic dermatitis, and psoriasis), (c) patient with a history of gastrointestinal surgery, (d) smokers with smoking index >400, (e) drinkers with daily ethanol intake ≥40 g for men (20 g for women) in the past five years or ≥80 g in past two weeks, and (f) use of systemic antibiotics, retinoids, corticosteroids or any other immunosuppressive therapy in the recent 6 months. The study was approved by the Medical Ethics Committee of the Third Affiliated Hospital of Chongqing Medical University, and written informed consent was obtained from all the participants at the time of recruitment.

### Patient Profiles

A structured questionnaire was used to collect general demographic variables (age, gender, ethnicity, height, and weight), medical history, previous or current medications, smoking habits, and alcohol intake. After 2 weeks of treatment, fasting venous blood collected from each patient was used to determine erythrocyte sedimentation rate (ESR) and white blood cells, and the serum was used to quantify protein levels of total IgE (#5613, MEIMIAN, Jiangsu, China), C-reactive protein (CRP, #E007462, 3ABio, Shanghai, China), and interleukin (IL)-6 (#E000482, 3ABio, Shanghai, China) by enzyme-linked immunosorbent assay (ELISA) following the instructions of the manufacturer from commercially-available kits. Autologous serum skin testing (ASST) was performed in all patients as previously described, and morphologically positive reactions (1+, 2+, or 3+) are assessed as auto-allergic. The urticarial activity score for 7 days (UAS7) at the time of patient recruitment and UAS at sample collecting were assessed for each patient according to the EAACI/GA^2^LEN/WAO/EDF guideline (Zuberbier et al., 2018).

### Fecal Sample Collection, DNA Extraction, and Sequencing

After 2 weeks of treatment with levocetirizine, approximately 5 g of fresh stool samples from each patient were collected in a sterile plastic cup and stored at −80°C immediately. The fresh stool was used to detect fecal calprotectin protein by a corresponding ELISA kit (#13732, MEIMIAN, Jiangsu, China). A QIAamp DNA stool mini kit (Qiagen, Hilden, Germany) was used to extract bacterial DNA from 200 ± 20 mg of feces according to the instructions of the manufacturer and a NanoDrop ND-1000 Spectrophotometer (Nucliber) to confirm the purity of extracted DNA (A260/280 ratio of 1.8); all extracted DNA samples were stored at −80°C until further analysis. To analyze bacterial colonies in feces, the amplification of the V3–V4 region of the 16S rRNA gene was performed using polymerase chain reaction (PCR) with bacterial universal primers 341F (5’-CCTACGGGNGGCWGCAG-3’) and 806R (5’-GGACTACHVGGGTATCTAAT-3’). All PCR reactions were performed in triplicate using a 50-μl mixture containing 1× Phanta^®^ Flash Master Mix (#P520, Vazyme, China), 10 μM primers, and 100 ng of template DNA. The amplification conditions were 98°C for 30 s (1 cycle), followed by 98°C for 10 s, 56°C for 5 s, 72°C for 5 s (30 cycles), and 72°C for 1 min (1 cycle). Amplicons were then recovered from 2% agarose gels and further purified using an AxyPrep DNA Gel Extraction Kit (Axygen Biosciences, CA, USA) and quantified by QuantiFluor-ST (Promega, USA) according to corresponding protocols. According to the instruction, purified amplicons were pooled in equimolar and paired-end sequenced (2 × 300) on an Illumina MiSeq platform (Illumina, San Diego, USA). The raw reads were deposited into the NCBI Sequence Read Archive (SRA) database (Accession Number: PRJNA809140).

### Bioinformatic Analysis and Statistical Tests

Paired-end raw reads with overlap were merged to tags, and tags were clustered to operational taxonomic units (OTUs) at 97% sequence similarity using UPARSE (version 7.1, http://drive5.com/uparse/). The taxonomy of each 16S rRNA gene sequence was analyzed using the RDP Classifier Algorithm (http://rdp.cme.msu.edu/) against the Silva (SSU128) 16S rRNA database, with a confidence threshold of 70%. QIIME and R packages (v3.2.0) from the free online Majorbio I-Sanger Cloud Platform (www.i-sanger.com) were applied to analyze sequencing data. Briefly, alpha diversity indices (Shannon index and Simpson index), evenness indices (Simpson even and Shannon even indices), and richness estimators (ACE index, Sobs index, and Chao 1 index) at OTU level were calculated using Mothur v.1.30.2. At the OTU level, partial least squares-discriminant analysis (PLS-DA) was applied to show the distance rank among the three groups. Non-metric multidimensional scaling (NMDS) was used to assess similarities in the microbial composition among three groups (beta diversity) using the R package, and stress <0.2 is generally expressed by the two-dimensional dot pattern of NMDS. Statistically, significant differences in the relative abundances of taxa were determined by Wilcoxon rank-sum test. Linear discriminant analysis (LDA) was used for assessing taxa responsible for the differences between groups. The gene function profiles of gut bacteria were predicted with a Phylogenetic Investigation of Communities by Reconstruction of Unobserved States (PICRUSt) analysis. Partial Pearson’s correlation controlling for age, sex, and BMI was calculated between the differential abundance at the genus level and clinical characteristics in the VGAM package. In addition, the continuous and categorical variables were presented as mean (M) ± standard deviation (SD) and percentage (%), respectively. The chi-square test or Student’s *t*-test was conducted to compare the demographic information and clinical characteristics of the two groups. A *P*-value <0.05 (two sides) was accepted as the cutoff for statistical significance, and a false discovery rate (FDR) was considered for the correction of the *P*-value.

## Results

### Characteristics of Patients

The demographic and clinical characteristics of the three groups are summarized in **Table 1**. They shared similarities in gender ratio, age, and BMI (*P* >0.05 for all variables). Compared with the CSU group, the RCSU group showed a remarkably longer course of disease (12.28 ± 5.49 *vs*. 7.00 ± 3.54 months, *P* <0.001), significantly higher value of UAS7 (21.52 ± 9.49 *vs*. 12.68 ± 4.67, *P* <0.001), UAS at sample collecting (3.60 ± 1.29 *vs*. 2.81 ± 0.89, *P* <0.05), ESR (14.20 ± 4.36 *vs*. 7.70 ± 2.40 mm/h, *P* <0.001), the serum protein level of CRP (8.97 ± 3.08 *vs*. 5.49 ± 1.98 mg/L, *P* <0.001), and IL-6 (8.71 ± 2.82 *vs*. 4.48 ± 1.69 pg/ml, *P* <0.001), and gut calprotectin (183.92 ± 98.41 *vs*. 125.49 ± 73.96 μg/g, *P* <0.05). The state of ASST and the total serum IgE did not differ in the two groups (*P* >0.05 for all variables).

**Table 1:** Comparison of demographic and clinical characteristics of the three groups.

Items	RCSU (n = 25)	CSU (n = 19)	HC (n = 19)
Sex (M/F)^#^	16/9	12/7	11/8
Age, (years)	34.42 ± 10.51	32.75 ± 10.83	31.45 ± 8.42
BMI, (kg/m^2^)	22.61 ± 1.50	21.98 ± 1.43	22.03 ± 1.36
Disease duration, (month)	12.28 ± 5.49***	7.00 ± 3.54	NA
UAS at sample collecting	3.60 ± 1.29*	2.81 ± 0.89	NA
UAS7	21.52 ± 9.49***	12.68 ± 4.67	NA
ASST: −/1+/2+/3+^#^	9/13/2/1	7/11/1/0	ND
Total serum IgE (μg/ml)	3.75 ± 1.32	3.07 ± 1.49	ND
ESR (mm/h)	14.20 ± 4.36***	7.70 ± 2.40	ND
Serum CRP (mg/L)	8.97 ± 3.08***	5.49 ± 1.98	ND
Serum IL-6 (pg/ml)	8.71 ± 2.82***	4.48 ± 1.69	ND
Calprotectin (μg/g)	183.92 ± 98.41*	125.49 ± 73.96	119.81 ± 68.78

BMI, body mass index; UAS, urticaria activity score; CSU, chronic spontaneous urticaria; RCSU, refractory CSU; Reference range of ESR, serum CRP, serum IL-6 is 0–15 mm/h, 0–4 mg/L, and 0–7 pg/ml. The assay ranges of IgE and Calprotectin ELISA kits are 0.1–4.5 μg/ml and 1–200 μg/g, respectively. Data were presented as mean ± SD except when indicated (#, cases). Chi-square or Student t-test was used for statistical differences between two groups. Compared with the CSU group,*P <0.05,**P <0.01,***P <0.001. ND, not detected; NA, not applicable.

### Sequencing Data

**Figure 1:**
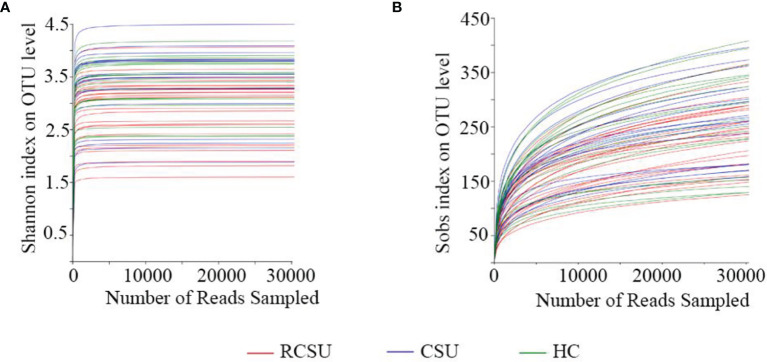
Analysis of 16s rRNA sequencing depth. Refraction curve analysis with Shannon **(A)** or Sobs index **(B)** on OTU level.

We obtained 3,163,836 (ranging from 75,262 to 139,363), 2,447,932 (ranging from 94,736 to 145,935), and 2,524,720 (ranging from 88,463 to 133,728) raw sequences from 25 fecal samples in the RCSU, 19 in the CSU, and 19 in the HC groups, respectively. After quality filtering and trimming, 1,491,943 (ranging from 40,244 to 88,336) valid sequences with a mean length of 419 bp, 926,357 (ranging from 56,506 to 75,638) with that 415 bp and 930,462 (ranging from 54,731 to 74,836) with that 416 bp were screened in the three groups, respectively. The reads from the three cohorts involved 1,344 OTUs, namely, 19 Phylum, 28 Classes, 68 Orders, 139 Families, 357 Genera, and 721 Species. The coverage estimator of the value of good was 99.99%. Clear asymptotes observed in refraction curve analyses with Shannon or Sobs index at OTU level indicates a near-complete sampling of the communities (**Figure 1**).

### Bacterial Diversity Analysis

**Figure 2:**
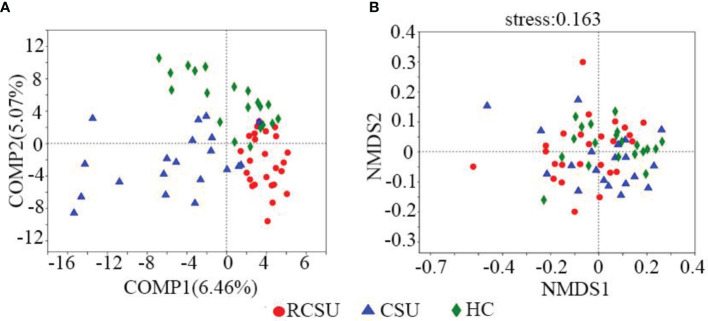
β Diversity analysis of gut microbial structure. **(A)** Comparison of distance rank on OTU level among three groups was shown by partial least squares-discriminant analysis (PLS-DA). **(B)** Similarities in the microbial composition on OTU level among three groups were presented by non-metric multidimensional scaling (NMDS). R, RCSU; N, CSU; C, HC.

As shown in **Table 2**, the RCSU and the CSU groups have comparable index values for ACE, Sobs, or Chao 1 at OTU level, demonstrating unchanged bacterial community richness in the two groups (*P* >0.05 for all variables). In comparison with the CSU group, Simpson estimator and index values for Shannon, Shannoneven, or Simpsoneven in the RCSU group were remarkably higher and lower, respectively (both *P* and corrected *P* <0.05 for all variables), which implies decreased alpha-diversity and evenness of fecal bacterial community in the RCSU patients. No significant difference in the alpha-diversity, richness, and evenness of the fecal bacterial community were observed between the CSU and the HC groups (*P* >0.05 for all variables). At the OTU level, distinguished cohorts were visualized by PLS-DA, and beta-diversity analyzed using the NMDS analysis based on unweighted UniFrac metrics also showed significant distinct clusters among the three cohorts (stress = 0.163, **Figures 2A** **, B**).

**Table 2:** α Diversity analysis in the three groups at OTU level.

	RCSU^1^	CSU^2^	HC^3^	*P(1vs.2)*	*P(2 vs.3)*
Sobs	225.36 ± 66.75	263.56 ± 71.903	285.32 ± 97.31	0.085	0.515
Chao	279.64 ± 87.01	311.22 ± 92.69	346.72 ± 127.11	0.266	0.416
Ace	282.13 ± 82.54	309.19 ± 89.23	338.63 ± 118.21	0.318	0.472
Shannon	2.88 ± 0.56	3.47 ± 0.56	3.27 ± 0.81	0.001*	0.478
Simpson	0.16 ± 0.09	0.08 ± 0.06	0.13 ± 0.13	0.003*	0.313
Simpsoneven	0.04 ± 0.02	0.07 ± 0.03	0.05 ± 0.02	0.005*	0.083
Shannoneven	0.53 ± 0.09	0.63 ± 0.08	0.58 ± 0.12	0.002*	0.283
Good coverage	0.99 ± 0.00	0.99 ± 0.00	0.99 ± 0.00	0.991	0.321
PD_ indexes	20.05 ± 5.13	22.59 ± 5.23	23.72 ± 7.17	0.120	0.644

Wilcox rank-sum test was used for statistical differences between groups. *Corrected P <0.05 for all variables.

### The Overall Distribution of Gut Microbiota

**Figure 3:**
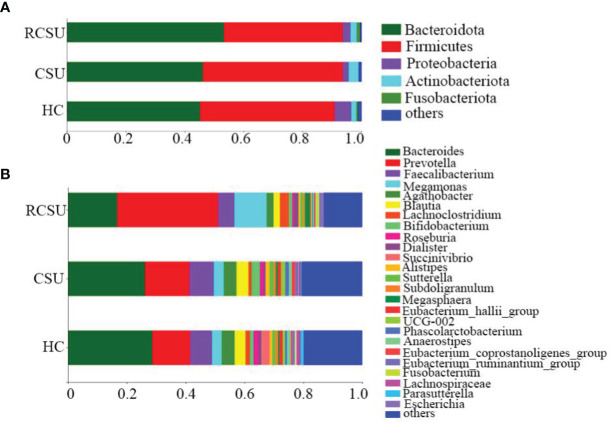
The composition and relative proportion of gut bacteria in the three groups on Phylum level **(A)** and Genus level **(B)**.

There was 208 shared bacterium in three groups on Genus level, with 23, 6, and 36 unique ones in the RCSU, the CSU, and the HC groups, respectively. More than 90% of fecal bacteria were Phyla *Bacteroidota* and *Firmicutes* in the three groups (**Figure 3A**). Regarding the relative abundance at the Genus level (**Figure 3B**), the top 8 taxa identified in the RCSU group included: *Prevotella* (34.27%), *Bacteroides* (16.72%), *Megamonas* (10.98%), *Faecalibacterium* (5.55%), *Lachnoclostridium* (3.13%), *Agathobacter* (2.34%), *Blautia* (2.07%), and *Megasphaera* (1.61%), and that in the CSU group were assigned to *Bacteroides* (26.16%), *Prevotella* (15.11%), *Faecalibacterium* (8.28%), *Blautia* (4.04%), *Agathobacter* (4.32%), *Megamonas* (3.38%), *Bifidobacterium* (2.76%), and *Alistipes*(1.41%), and that in the HC group were *Bacteroides* (28.68%), *Prevotella* (12.82%), *Faecalibacterium* (7.40%), *Agathobacter* (4.41%), *Blautia* (3.62%), *Megamonas* (3.34%), *Succinivibrio* (2.84%), and *Roseburia* (1.67%).

### Community Structure Differences

At the phylum level, no significant differences in the relative abundance of the bacterium were detected among the three groups (*P* >0.05 for all variables). Differences in the relative abundances of the top 30 genera composition of the intestinal microflora in the three groups were further analyzed using the Wilcox rank-sum test. Results showed that the RCSU group displayed a significant increase in *Prevotella* (34.28 ± 33.28 *vs*. 15.12 ± 22.27, *P* <0.05), *Megamonas* (10.99 ± 15.48 *vs*. 3.38 ± 6.01, *P* <0.05), and *Escherichia* (1.16 ± 1.85 *vs*. 0.11 ± 0.25, *P* <0.05), while members of the *Blautia* (2.08 ± 2.34 *vs*. 4.04 ± 2.72, *P* <0.05), *Alistipes* (0.59 ± 0.97 *vs*. 1.40 ± 1.48, *P* <0.05), *Anaerostipes* (0.25 ± 0.37 *vs*. 0.95 ± 1.11, *P* <0.01), and *Lachnospira* (0.36 ± 0.48 *vs*. 0.94 ± 1.15, *P* <0.05) were relatively decreased compared to the CSU group (**Table 3**). However, only *Escherichia* remained significantly altered after correcting the *P*-values. No significant differences in genera bacterium were found between the CSU and the HC groups (*P* >0.05 for all variables)

**Table 3:** Relative abundance of genera bacterium among the three groups.

Items	RCSU	CSU	HC
*Bacteroides*	16.72 ± 19.30	26.16 ± 26.87	28.69 ± 26.22
*Prevotella*	34.28 ± 33.28*	15.12 ± 22.27	12.82 ± 15.32
*Faecalibacterium*	5.54 ± 4.72	8.28 ± 5.07	7.40 ± 5.58
*Megamonas*	10.99 ± 15.48*	3.38 ± 6.01	3.34 ± 6.11
*Agathobacter*	2.34 ± 2.80	4.32 ± 3.99	4.41 ± 4.61
*Blautia*	2.08 ± 2.34*	4.04 ± 2.72	3.62 ± 2.72
*Bifidobacterium*	1.09 ± 1.17	2.76 ± 4.12	2.08 ± 2.12
*Roseburia*	0.61 ± 0.64	1.27 ± 1.56	1.67 ± 1.57
*Alistipes*	0.59 ± 0.97*	1.40 ± 1.48	0.91 ± 1.51
*Anaerostipes*	0.25 ± 0.37**	0.95 ± 1.11	1.19 ± 1.81
*Parabacteroides*	0.55 ± 0.94	0.88 ± 0.70	0.73 ± 0.51
*Lachnospira*	0.36 ± 0.48*	0.94 ± 1.15	0.76 ± 0.88
*Dorea*	0.72 ± 1.28	0.74 ± 0.43	0.51 ± 0.58
*Lactobacillus*	0.57 ± 1.64	0.78 ± 0.52	0.63 ± 0.77
*Escherichia*	1.16 ± 1.85***^#^	0.11 ± 0.25	0.11 ± 0.17

Relative abundances of genera bacterium were presented as M ± SD, and differences were assessed by Wilcoxon rank-sum test among the RCSU and the CSU groups or the CSU and the HC groups. Compared with CSU group,*P <0.05, **P <0.01, ***P <0.001, ^#^corrected P <0.001.

**Figure 4:**
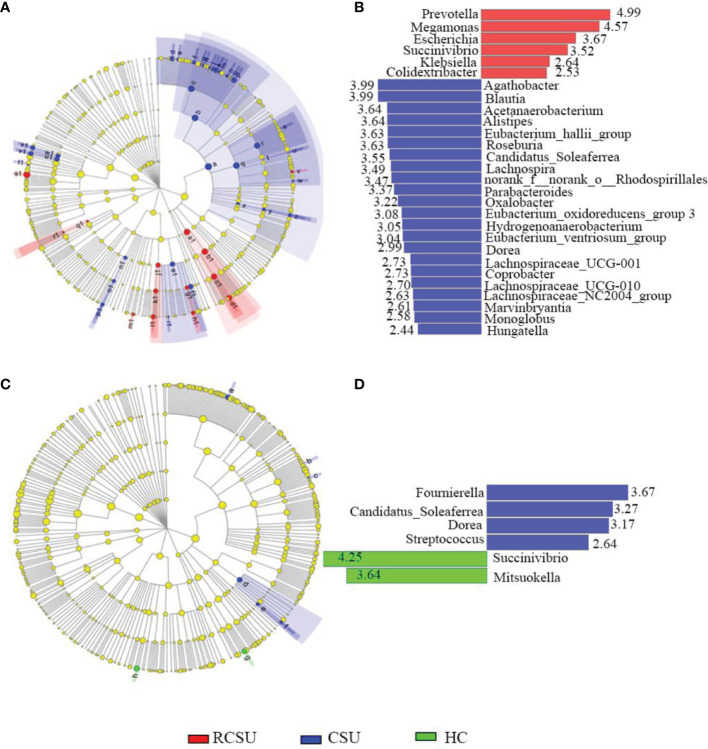
Differently abundant taxa identified using LEfSe analysis among groups. Distribution of differently abundant taxa from phylum to genera levels between the RCSU and the CSU groups **(A)** and between the CSU and the HC groups **(C)**. LDA showing the impact of different species on the difference between the RCSU and the CSU groups **(B)**, and between the CSU and the HC groups **(D)**, and visualization of only taxa meeting an LDA ≥2.

In addition, the application of the LefSe method identified a total of 42 features with significantly different abundances from phylum to genera levels between the RCSU and the CSU groups (LDA score >2 with *P <*0.05, **Figure 4A**). Specifically, fecal microbiota of the RCSU patients was differently enriched with genera *Prevotella*, *Megamonas*, *Escherichia*, *Succinivibrio*, *Klebsiella*, and *Colidextribacter* (*P <*0.05 for all variables), whereas the CSU group was enriched with genera *Agathobacter*, *Blautia*, *Acetanaerobacterium*, *Alistipes*, *and Eubacterium_hallii_group*, etc. (*P <*0.05 for all variables, **Figure 4B**). Contrastly, between CSU and HC groups, LefSe only identified 7 distinctive features with more genera *Succinivibrio* and *Mitsuokella* in the latter group (*P <*0.05 for all variables, **Figures 4C** **, D**).

### Relationship With Clinical Characteristics

**Figure 5:**
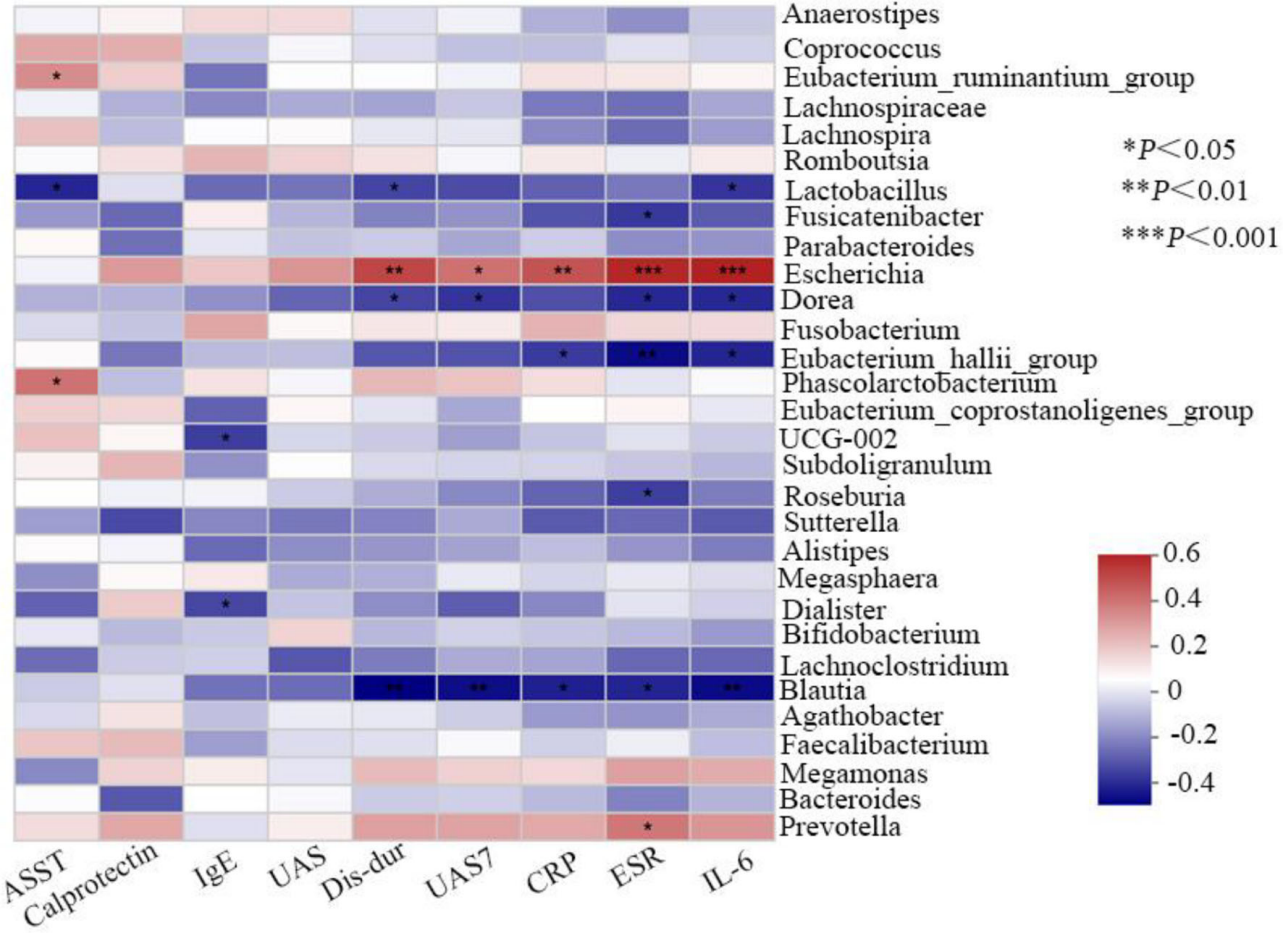
Correlation analysis of gut bacteria and related clinical indicators.

Finally, analyses of the relationship between observed genera in all the patients and clinical characteristics were conducted. Variance inflation factor (VIF) of clinical indicator more than 10 was considered to influence subsequent correlation analysis as the existence of multicollinearity between clinical factors. By inspection, VIF of disease duration, UAS at sample collecting, UAS7, ASST, serum IgE, ESR, IL-6, CRP, and calprotectin was 7.29, 3.07, 5.81, 2.11, 1.48, 6.11, 1.54, 6.10, and 1.86, respectively. As the correlation Heatmap graph shows (**Figure 5**), the abundance of the genera *Escherichia* demonstrated a strong positive correlation with ESR (*R* = 0.532, *P* <0.001) and serum IL-6 (*R* = 0.545, *P* <0.001), moderate with disease duration (*R* = 0.461, *P* <0.01) and serum CRP (*R* = 0.433, *P* <0.01), and weak with UAS7 (*R* = 0.368, *P* <0.05). In addition, *Prevotella* is also positively associated with ESR (*R* = 0.453, *P*<0.05). Contrastly, ESR (*R* = −0.418, *P* <0.01), UAS7 (*R* = −0.411, *P* <0.05), serum IL-6 (*R* = −0.417, *P* <0.01), and CRP (*R* = −0.377, *P* <0.05) all were negative with that of genera *Blautia*. Although not strong, negative associations between the systemic inflammation indicators and *Dorea*, *Lactobacillus*, *Eubacterium_hallii_group*, and *Roseburia* were shown (*R* <0, *P* <0.05 for all variables). However, no correlation was found between the content of gut calprotectin and the observed genera.

## Discussion

Clarifying the pathophysiology of nsAH-resistance in CSU is a continuing need, and efforts are in progress (Huilan et al., 2015; Kaplan, 2018). IgE and/or IgG antibodies are the best-known triggers to cause mast cell degranulation within tens of minutes and production of inflammation mediators within a few hours, which are considered critical biological events in CSU pathogenesis (Kolkhir et al., 2017; Bracken et al., 2019). However, serum levels of IgE/IgG, histamine released from mast cells degranulation, and antihistamine therapy efficacy are not completely parallel in CSU patients. Other than IgE or IgG, a plethora of stimuli such as toll-like receptor (TLR) ligands, cytokines, and chemokines can also directly activate mast cells to produce inflammatory mediators without degranulation; however, this way may exert a synergic or antagonistic effect on IgE/IgG-mediated mast cell degranulation, indicating the potential importance of non-IgE/IgG-mediated mast cell activation in the aggravation or mitigation of inflammation in allergic diseases including CSU (Yu et al., 2016; Varricchi et al., 2019). In the current study, a comparable level of total serum IgE in CSU with or without nsAH-resistance further implies the presence of other potential factors interfering with IgE-mediated histamine release (Altrichter et al., 2021). In addition, the evidence that nsAH-resistant CSU patients showed more severe disease activity and intestinal (measured by stool calprotectin) or systemic proinflammatory response argue for the pivotal role of non-immunity-mediated inflammation in the occurrence of nsAH resistance.

The current study was the first attempt to profile the gut microbiota and determine its correlation with systemic inflammation in CSU patients with nsAH-resistance. Gut microbiota disturbance was widely proved to be involved in the onset or severity of chronic inflammatory skin diseases (ISD) such as atopic dermatitis, psoriasis, and acne vulgaris (O'Neill et al., 2016). Gut microbiota profiles between chronic urticaria or CSU and healthy volunteers were recently characterized in related studies (Lu et al., 2019; Wang et al., 2020; Wang et al., 2021; Zhang et al., 2021), and decreased alpha diversity of the bacterial community was supposed to be of great importance in chronic urticaria. In the current study, lower bacterial alpha-diversity and evenness in CSU patients with nsAH resistance than those without further underline the correlation between enteric dysbacteriosis and CSU severity. Although the causal relationship remains controversial, decreased microbiota diversity may partly link with the etiology or severity of CSU by disrupting Treg cell-mediated intestinal mucosal immunological tolerance as it could cause increased susceptibility of allergic diseases (Kurashima et al., 2013; Kim et al., 2016). Regarding beta diversity measured with spatial distance among CSU individuals with or without nsAH resistance and healthy populations, PLS-DA showed that the fecal samples from any cohort were able to be clustered independently, which is partly consistent with findings from other studies (Wang et al., 2020; Zhang et al., 2021) and is not surprising as subjects were carefully screened and grouped.

Increasing evidence suggested significant differences in gut microbiota composition between urticaria patients and healthy individuals. Contrasting to that assayed by 16s rRNA massive sequencing, significantly lower amounts of *Akkermansia muciniphila*, *Faecalibacterium prausnitzii*, *Clostridium leptum*, *Lactobacillus*, and *Bifidobacterium* in fecal samples from patients with chronic urticaria versus the healthy measured by real-time polymerase chain reaction were firstly reported in the team of Nabizadeh (Nabizadeh et al., 2017; Rezazadeh et al., 2018). After that, compared with healthy controls, Lu et al., found that chronic urticaria patients have a higher abundance of genera *Escherichia*, and lower *Faecalibacterium*, *Prevotella*, and *Bacteroides* in feces (Lu et al., 2019). In another three studies focusing on comparisons between CSU patients with unknown nsAH resistance and healthy population, relatively more abundant genera *Turicibacter* and *Lachnobacterium*, less abundant genera *Faecalibacterium*, *Bacteroides*, *Bifidobacterium*, *Megamonas*, *Megasphaera*, *Phascolarctobacterium*, and *Dialister* were observed in stool of patients (Wang et al., 2020; Wang et al., 2021; Zhang et al., 2021). Differences in numbers of participants, gender ratios, dietary habits, and previous therapeutics among studies may lead to partially inconsistent conclusions. CSU patients without nsAH resistance and HC individuals showed almost unchanged genera bacterium among our participants from Southwest China. However, relative increased abundance of genera *Prevotella*, *Megamonas*, and *Escherichia*, whereas the relative reduced abundance of genera *Blautia*, *Alistipes*, *Lachnospira*, and *Anaerostipes* were detected in CSU individuals with nsAH resistance when compared with nsAH-responsive CSU cases. These results suggest that uncovering the variability of gut flora by sub-classifying the patients according to certain clinical characteristics is warranted. In addition, the evidence that correction of gut dysbiosis in adult RCSU or CSU patients using adjunct therapy with probiotics only achieved limited efficacy based on the UAS7 score implies probiotics supplementation may not always work (Nettis et al., 2016; Atefi et al., 2021). It may be reasonable as gut *Bifidobacterium* and *Lactobacillus* were not reduced in all patients, as was the case in the current study.

Chronic low-level pro-inflammation phenotype is usually considered to contribute to CSU pathogenesis. Therefore, what is the relationship between the occurrence of nsAH resistance and altered intestinal flora in CSU from the inflammation perspective? Although studies focusing on enterobacteria and nsAH resistance in allergic diseases are not available, linking our results with previous related reports (Lu et al., 2019; Wang et al., 2020; Wang et al., 2021; Zhang et al., 2021), CSU patients with nsAH resistance are thought to be the subgroup with more severe systemic inflammation that partly related to specific enterobacteria. Specifically, we showed that the abundance of genera *Escherichia* and *Prevotella* negatively correlated with systemic not intestinal inflammation. Biologically, it was reported that *Escherichia* exposure directed mast cell responses toward Th2-dependent inflammation and away from IgE-mediated effects by reducing Fc receptor for IgE (FcϵRI) expression and FcϵRI -mediated mast cell degranulation (Kulka et al., 2006), which may further support the finding that CSU patients with more severe systemic inflammation have higher susceptibility of nsAH resistance. Recently, *Prevotella* was frequently identified as a proinflammatory gut bacterium in some diseases (Hofer, 2014; Li et al., 2019; Cao et al., 2020; Zhou et al., 2020). On the contrary, the result that genera *Blautia*, *Dorea*, *Lactobacillus*, *Eubacterium_hallii_group* and *Roseburia* negatively correlated with systemic inflammation in our studies partly coincides with previous findings that *Blautia* and *Lactobacillus* inhibited inflammation response by suppressing the production of short-chain fatty acid in the gut (Benítez-Páez et al., 2020). In addition, recent evidence that compound probiotic intervention alleviated inflammatory response mediated by the *Escherichia* challenge may also provide the rationale to correct gut dysbiosis in nsAH-resistant CSU patients using probiotics supplementation (Sun et al., 2020; Liu et al., 2022), but more clinical studies are required to confirm the efficacy.

## Conclusion

Limitations in the existence of the current study include: 1) the causal relationships among specific enterobacteria, systemic inflammation, and nsAH resistance in CSU failed to be adequately explained as with all case-controlled clinical studies, 2) assessments of intestinal flora, and systemic inflammation before and after nsAH treatment are further required to determine the effect of poor treatment response on related changes, 3) within-subject verification of related biomarkers and replication procedures in larger study populations from multicenter are lacking, and assessment of systemic inflammation in the healthy control group in parallel is deficient, 4) nsAHs other than levocetirizine such as ebastine and olopatadine were not included in this study. However, the present study indicates that pro-inflammation phenotype relating to enteric dysbacteriosis features nsAH resistance in CSU patients, which provides clues for future microbial-based or anti-inflammatory therapies on nsAH resistant CSU.

## Data Availability Statement

The data presented in the study are deposited in the NCBI Sequence Read Archive (SRA) database, accession number: PRJNA809140.

## Ethics Statement

The studies involving human participants were reviewed and approved by the Medical Ethics Committee of the Third Affiliated Hospital of Chongqing Medical University. The patients/participants provided their written informed consent to participate in this study.

## Author Contributions

FH and BC designed the study. YS wrote the manuscript. ZY and XY collected serum samples. YS and KD carried out the experiments and analyzed the data. All authors listed have made a substantial, direct, and intellectual contribution to the work and approved it for publication.

## Funding

This work was funded by grants from the National Natural Science Foundation of China (82003337), the China Postdoctoral Science Foundation (2020M683268), the Chongqing Natural Science Foundation (cstc2020jcyj-bshX0023), the Chongqing Excellence Programme (cstc2021ycjh-bgzxm0291), the Postdoctoral Foundation of Chongqing Medical University (R9001) and the Funding for Key Disciplines of Third Affiliated Hospital of Chongqing Medical University (ZK201902).

## Conflict of Interest

The authors declare that the research was conducted in the absence of any commercial or financial relationships that could be construed as a potential conflict of interest.

## Publisher’s Note

All claims expressed in this article are solely those of the authors and do not necessarily represent those of their affiliated organizations, or those of the publisher, the editors and the reviewers. Any product that may be evaluated in this article, or claim that may be made by its manufacturer, is not guaranteed or endorsed by the publisher.
